# Beyond genotype‐4: Direct‐acting antiviral agents in patients with chronic hepatitis C infection from the Eastern Province of Saudi Arabia

**DOI:** 10.1002/hsr2.1795

**Published:** 2024-01-05

**Authors:** Mona H. Ismail

**Affiliations:** ^1^ College of Medicine at Imam Abdulrahman bin Faisal University Dammam Saudi Arabia; ^2^ Department of Internal Medicine, Division of Gastroenterology King Fahd Hospital of the University Al Khobar Saudi Arabia

**Keywords:** direct‐acting antiviral agent, hepatitis C virus, Saudi Arabia, sustained virologic response

## Abstract

**Background and Aims:**

Direct‐acting antiviral agents (DAAs) have revolutionized the treatment of patients with chronic hepatitis C virus (HCV) infection, resulting in a high sustained virologic response (SVR) rate. However, the published data from the Eastern Province of Saudi Arabia are limited to small patient groups and specific DAAs used for patients with genotype‐4.(GT‐4). This study aimed to investigate the effectiveness and safety of DAAs for treating HCV infection in Saudi Arabia in a real‐life setting.

**Methods:**

This retrospective study from January 2015 to December 2019 included all HCV‐infected patients who received DAAs at a tertiary university hospital in Saudi Arabia. Baseline characteristics and laboratory data were collected from health records, including HCV RNA level, genotype, and presence of liver cirrhosis or steatosis. The primary outcome was undetectable HCV RNA at 12 weeks posttreatment (SVR12). Results were stratified based on different DAAs and HCV genotypes. Treatment‐related adverse events were recorded. Statistical analyses were performed using SPSS version 25.0.

**Results:**

Of the 117 patients included, 43.2% had advanced fibrosis or cirrhosis, and the majority (90.6%) were treatment‐naïve. The mean age was 50.1 ± 15.5 years, with 57.3% females. The most common genotype was GT‐4 (44.4%), followed by GT‐1 (40.2%). Most patients (64.3%) received sofosbuvir and daclatasvir ± ribavirin, while the remaining patients received various DAAs. Overall, 98.3% of the patients achieved SVR12. The therapy was well tolerated, with fatigue and headache being the most common side effects.

**Conclusions:**

Treatment with DAAs is highly effective across different genotypes and various DDA regimens in the real world for treating HCV infection in the Eastern Province of Saudi Arabia, contributing to improved patient outcomes and the overall goal of HCV elimination.

## Key points

- **What is Known**: Direct‐acting antiviral agents (DAAs) have revolutionized the treatment of chronic hepatitis C virus (HCV) infection, resulting in high sustained virologic response (SVR) rates globally. Published data from Saudi Arabia are limited to small patient groups and specific DAAs used for patients with genotype‐4.
- **What is New**: This study provides comprehensive data on the effectiveness and safety of DAAs in a real‐world setting, including diverse HCV genotypes and various DAA regimens and their association with comorbid conditions.
- **Clinical Implications**: The high SVR12 rates observed with DAAs in this study from Saudi Arabia align with the global efforts to eliminate HCV infection and improve patient outcomes.

## 1 INTRODUCTION

Chronic hepatitis C virus (HCV) infection is a significant global health issue, affecting approximately 71 million people worldwide.^1^ In Saudi Arabia, the prevalence of HCV infection is estimated to be 1.4%, corresponding to around 400,000 individuals with the disease.^2^ Chronic HCV infection can lead to severe complications such as liver cirrhosis, liver failure, and hepatocellular carcinoma, making it a major cause of liver‐related morbidity and mortality on a global scale.^1^, ^3^

Historically, the standard treatment for chronic HCV infection involved interferon (IFN)‐based therapy, which exhibited low response rates, significant side effects, and poor tolerability.^4^ However, introducing direct‐acting antiviral agents (DAAs) has transformed the treatment landscape, resulting in high rates of sustained virologic response (SVR) and improved clinical outcomes. DAAs are highly effective in achieving SVR, defined as undetectable HCV RNA at 12 weeks posttreatment, while demonstrating fewer side effects than IFN‐based therapy.^5^

Despite the well‐established and widely accepted efficacy of DAA therapy in treating hepatitis C infection, it is essential to publish studies that reaffirm its effectiveness in the specific context of Saudi Arabia due to unique demographic and epidemiological factors. Although several studies have reported high SVR rates with DAA therapy in Saudi Arabia, they have been limited by small sample sizes ranging from 34 to 213 and the utilization of specific DAAs. For example, some studies focused on the combination of simeprevir or daclatasvir with sofosbuvir in patients infected with genotype‐4 (GT‐4),^6^ while others have examined the use of ledipasvir/sofosbuvir in GT‐4‐infected patients with advanced liver fibrosis and decompensated cirrhosis,^7^ or paritaprevir/ritonavir/ombitasvir plus dasabuvir in patients with GT‐1 and GT‐4 with severe kidney disease.^8^ Additionally, there is limited data regarding the effectiveness and safety of various DAAs in patients with diverse HCV genotypes, specifically from the Eastern Province of Saudi Arabia. Therefore, the aim of this study was to investigate the effectiveness and safety of DAAs in treating HCV infection in the Eastern Province of Saudi Arabia.

## 2 MATERIALS AND METHODS

### 2.1 Study design and patients

This retrospective study included consecutive patients aged ≥18 years with positive HCV RNA by polymerase chain reaction (PCR) who attended the Hepatology clinics at King Fahd University Hospital (Al‐Khobar, Saudi Arabia) between January 2015 and December 2019. Patients were treatment‐naïve or treatment‐experienced (previous pegylated [PEG‐IFN]/ribavirin (RBV) or DAAs). We excluded patients with hepatitis B or human immunodeficiency virus coinfection, active substance or alcohol abuse, and hepatobiliary malignancy.

### 2.2 Clinical and laboratory assessment

The baseline characteristics were obtained by carefully reviewing the patient's electronic health records and our hospital's predefined HCV treatment protocol. We collected information on the patient's age, sex, nationality, body mass index (BMI), comorbidities, and previous treatment with HCV antiviral therapy. We also collected baseline laboratory values such as hemoglobin level, platelet count, alanine aminotransferase, aspartate aminotransferase, γ‐glutamyl transferase, bilirubin, serum albumin level, serum creatinine, prothrombin time, international normalized ratio (INR). Patients were evaluated in the clinic at least twice while on therapy. The diagnosis of liver cirrhosis was based on the combination of clinical and radiologic evidence of end‐stage liver disease (e.g., ascites, collateral vessels, and splenomegaly) and a fibroscan score of ≥14.6 kPa.

### 2.3 Assessment of liver fibrosis and steatosis

Liver stiffness measurement (LSM) by transient elastography and controlled attenuation parameter (CAP) (502 Touch; Echosens) was performed by an experienced operator (M. H. I.), who performed more than 2000 fibroscans, according to the manufacturer's recommendations before treatment initiation. LSM with the lowest variability <30% (interquartile range [IQR]/median) was selected for analysis. The results were considered unreliable if the number of valid attempts was <10, the success rate was <60%, or the IQR/median was >30%. While the presence of hepatic steatosis was based on CAP > 248 dB/m.^9^

### 2.4 Measurement of HCV RNA and genotyping

Serum HCV‐RNA levels and HCV genotype were measured using an automated real‐time PCR (RT‐PCR technology) assay (Abbott Real‐Time m2000). Serum HCV‐RNA levels were measured at baseline and 12 weeks after finishing therapy, and the lower limit of quantification (LLOQ) was 15 IU/mL.

### 2.5 Treatment regimens

Patients were treated with different DAAs based on the clinical characteristics of each patient and the DAA treatment available from the Saudi Ministry of Health following the national and international guidelines.^10^, ^11^, ^12^ The different DAAs used were as follows: one tablet of sofosbuvir 400 mg daily, one tablet of the combination of sofosbuvir 400 mg plus daclatasvir 60 mg daily, or one tablet of the combination of ledipasvir 90 mg and sofosbuvir 400 mg daily, or the combination of ombitasvir 12.5 mg/paritaprevir 75 mg/ritonavir 50 mg twice daily and dasabuvir 250 mg daily, or three tablets of glecaprevir 300 mg/pibrentasvir 120 mg daily, or one tablet of elbasvir 50 mg and grazoprevir 100 mg daily, or one tablet of sofosbuvir 400 mg/velpatasvir 100 mg daily. The treatment was given for 12 weeks ± RBV. The dose of RBV was 1000 mg daily for those <75 kg in weight and 1200 mg for those >75 kg, given in two divided doses for 12 weeks. While patients with cirrhosis were initially started on 600 mg daily, which was increased according to the patient's tolerance. The addition of RBV^13^ was based on the stage of liver fibrosis if the patient was treatment‐experienced or had one of the following criteria: total serum bilirubin >1.2 mg/dL, serum albumin <3.5 g/dL, INR >1.2, or platelets <150,000/L.

### 2.6 Effectiveness of treatment and safety evaluation

The effectiveness of therapy was assessed by the proportion of patients with a serum HCV RNA level LLOQ (15 IU/mL) 12 weeks after completion of treatment (SVR12). Furthermore, patients were classified as nonresponders if their HCV RNA was detected 12 weeks after finishing therapy. The safety evaluation was the adverse event reported by the patients during follow‐up, and adherence to the treatment regimen was collected at each visit.

### 2.7 Statistical analyses

Categorical data are presented as frequencies and percentages, while continuous data are presented as the mean and standard deviation (SD) or the median and IQR based on the normality of the data. The demographic characteristics, clinical data, and laboratory findings of the patients with and without SVR were compared. The *χ* ^2^ or Fisher's exact test was used to examine differences in categorical variables, whereas Student's *t*‐test or the Mann–Whitney test was used to analyze differences in quantitative variables. All *p* values were two‐tailed, and a *p* < 0.05 was considered statistically significant. The data analysis was performed using SPSS version 25.0 (IBM Corp.).

## 3 RESULTS

### 3.1 Baseline characteristics of patients

A total of 117 patients met our inclusion criteria. The mean age was 50.1 ± 15.5 years, of which 57.3% were females and 81.2% were Saudi nationals. The most common comorbidities were nonalcoholic fatty liver disease (NAFLD) (53.0%) and dyslipidemia (14.5%). Most patients (90.6%) were treatment‐naïve, and only 11 (9.4%) were treatment‐experienced (Table 1).

**Table 1:** Baseline clinical characteristics of the patients by treatment response (*N* = 117).

	All patients (117)	Sustained virological response	
		No (*n* = 2)	Yes (*n* = 115)
Age (years)	50.1 ± 15.5	28.0 ± 7.1	50.5 ± 15.3
Sex			
Male	50 (42.7)	1 (50.0)	49 (42.6)
Female	67 (57.3)	1 (50.0)	66 (57.4)
Nationality			
Saudi	95 (81.2)	2 (100.0)	93 (80.9)
Non‐Saudi	22 (18.8)	0 (0.0)	22 (19.1)
BMI (kg/m^2^)	30.6 ± 8.0	26.6 ± 5.8	30.7 ± 8.1
Comorbidity			
NAFLD	62 (53.0)	0 (0.0)	62 (53.9)
Dyslipidemia	17 (14.5)	0 (0.0)	17 (14.8)
Renal disease	4 (3.4)	0 (0.0)	4 (3.5)
Liver transplant	1 (0.9)	0 (0.0)	1 (0.9)
Other diseases^a^	2 (1.7)	0 (0.0)	2 (1.7)
Laboratory tests			
Hb (g/dL)	13.3 ± 2.2	13.1 ± 0.3	13.3 ± 2.2
ALT (IU/L)	72.4 ± 66.9	114.5 ± 79.9	71.6 ± 66.8
AST (IU/L)	59.2 ± 59.2	136.0 ± 91.9	57.9 ± 58.2
GGT (IU/L)	96.9 ± 92.7	294.5 ± 102.5	93.5 ± 89.2
Bilirubin (mg/dL)	0.84 ± 1.08	1.10 ± 0.71	0.83 ± 1.08
Albumin (g/dL)	3.56 ± 0.48	2.85 ± 0.50	3.57 ± 0.47
Platelets (×10^9^/L)	208.1 ± 87.9	124.5 ± 81.3	209.6 ± 87.6
Creatinine (mg/dL)	1.09 ± 1.45	0.69 ± 0.18	1.10 ± 1.46
Prothrombin time (s)	13.3 ± 1.6	13.7 ± 0.6	13.3 ± 1.6
INR	1.06 ± 0.14	1.15 ± 0.07	1.06 ± 0.14
Presence of ascites	2 (1.7)	0 (0.0)	2 (1.7)
HCV treatment‐experienced			
PEG‐IFN/RBV	6 (5.2)	0 (0.0)	6 (5.2)
SOF ± RBV	3 (2.6)	0 (0.0)	3 (2.6)
Telaprevir	2 (1.7)	0 (0.0)	2 (1.7)

*Note*: Data are presented as numbers (percentages) or means ± standard deviations.

Abbreviations: ALT, alanine transaminase; AST, aspartate transaminase; BMI, body mass index; GGT, γ‐glutamyl transferase; Hb, hemoglobin; HCV, chronic hepatitis C virus; INR, international normalized ratio; NAFLD, nonalcoholic fatty liver disease; PEG‐IFN, pegylated interferon‐α; RBV; ribavirin, SOF; sofosbuvir.

^a^ Other diseases were systemic lupus erythematosus and hemophilia.

The most common genotype was GT‐4 (44.4%), followed by GT‐1 (40.2%). Based on the Fibroscan results, the median LSM was 13.8 ± 14.3 kPa, the median CAP was 227.0 ± 76.3 dB/m, and advanced fibrosis or cirrhosis was seen in 43.2% (Table 2).

**Table 2:** HCV virology and Fibroscan results of patients by treatment response (*N* = 117).

	All patients	Sustained virological response (SVR12)	
	*N* = 117	No (*n* = 2)	Yes (*n* = 115)
HCV‐PCR (IU/mL) (log‐transformed)	13.4 ± 1.7	14.0 ± 1.3	13.4 ± 1.7
HCV genotype			
1	47 (40.2)	0 (0.0)	47 (40.2)
2	10 (8.5)	0 (0.0)	10 (8.5)
3	4 (3.4)	0 (0.0)	4 (3.4)
4	52 (44.4)	2 (0.0)	50 (43.5)
Mixed^a^	4 (3.4)	0 (0.0)	4 (3.4)
TE			
LSM, med (kPa)	13.8 ± 14.3	11.2 ± 9.4	13.9 ± 14.4
CAP, med (dB/m)	227.0 ± 76.3	189.0 ± 82.0	227.7 ± 76.4
LSM by TE (kPa)			
F0–F2 (<9.5)	62 (56.9)	1 (50.0)	61 (57.0)
F3 (9.5–14.4)	21 (19.3)	0 (0.0)	21 (19.6)
F4 (≥14.5)	26 (23.9)	1 (50.0)	25 (23.4)
Hepatic steatosis by CAP (%)			
Mild (<10)	1 (50.0%)	28 (27.5%)	29 (27.9%)
Moderate (10–30)	0 (0.0%)	62 (60.8%)	62 (59.6%)
Severe (>30)	1 (50.0%)	12 (11.8%)	13 (12.5%)

*Note*: Data are presented as numbers (percentages) or means ± standard deviations.

Abbreviations: CAP, controlled attenuation parameter; GT, genotype; HCV, chronic hepatitis C virus; LSM, liver stiffness measurement; med, median; PCR, polymerase chain reaction; TE, Transient elastography.

^a^ Mixed genotypes comprised three patients with GT‐1 and GT‐4 and a patient with GT‐3 and GT‐4.

### 3.2 Effectiveness of treatment with DAA therapy

**Figure 1:**
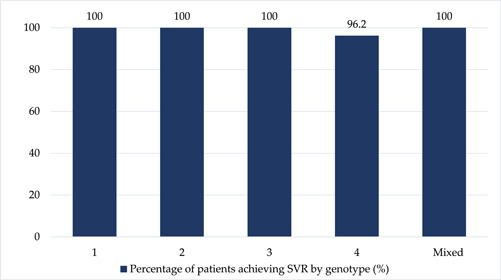
Percentage of patients who achieved sustained virological response by genotype.

Overall, the SVR12 rate was high at 98.3% (115 out of 117 patients), with all patients completing the 12 weeks of DAA therapy. Notably, SVR12 was achieved in 100% of patients with HCV GTs 1, 2, 3, and mixed genotypes. However, in patients with GT‐4 infection, the SVR12 rate was slightly lower, at 96.2% (Figure 1).

#### 3.2.1 Subgroup analysis of patients receiving sofosbuvir and daclatasvir

The SVR12 rates for patients treated with sofosbuvir and daclatasvir ± RBV were remarkably high, reaching 97.4%. However, it is noteworthy that only two patients experienced relapse after treatment with sofosbuvir and daclatasvir. Both patients were young (23 and 33 years old, respectively), treatment‐naïve, and one of them had liver cirrhosis (Table 3).

**Table 3:** Response to current antiviral therapy by SVR12 (*N* = 117).

Type of DAAs received	Sustained virological response	
	No (*n* = 2)	Yes (*n* = 115)
SOF and DCV ± RBV	2 (100.0)	74 (64.3)
SOF and RBV^a^	0 (0.0)	18 (15.7)
LDV/SOF	0 (0.0)	12 (10.4)
GLE/PIB	0 (0.0)	5 (4.3)
PrO and dasabuvir	0 (0.0)	4 (3.47)
EBR/GZR	0 (0.0)	1 (0.87)
SOF/VEL	0 (0.0)	1 (0.87)

*Note*: Data are presented as numbers (percentages).

Abbreviations: DAA, direct‐acting antiviral agent; DAC, daclatasvir; GLE/PIB, glecaprevir/pibrentasvir; GZR/EBR; elbasvir and grazoprevir; LDV, ledipasvir; PrO, paritaprevir/ritonavir/ombitasvir; RBV, ribavirin; SOF, sofosbuvir; SVR, sustained virological response; VEL, velpatasvir.

^a^ One patient received pegylated interferon‐α with this combination.

#### 3.2.2 Subgroup analysis of patients receiving other DAAs

Among the patients who received the remaining DAAs, SVR12 rates were observed to be 100%. Specifically, 18 patients received sofosbuvir ± RBV (with one patient receiving PEG‐IFN), 12 patients received ledipasvir/sofosbuvir, five patients were treated with glecaprevir/pibrentasvir, and three patients received either ombitasvir/paritaprevir/ritonavir and dasabuvir, elbasvir/grazoprevir, or sofosbuvir/velpatasvir (Table 3). Due to the high SVR12 rates across almost all patients, the non‐SVR12 group was too small to statistically identify differences in patients' characteristics between the two groups.

### 3.3 Safety of treatment received and adverse events

Overall, the treatment was well tolerated, and no patients discontinued therapy during the treatment period. Out of the total patients, 11 (9.4%) patients reported adverse events. Fatigue was reported by five patients (4.3%), with four of them receiving sofosbuvir and daclatasvir and one patient receiving paritaprevir/ritonavir‐ombitasvir. Four patients (3.4%) experienced headaches, including one patient treated with paritaprevir/ritonavir‐ombitasvir, two patients receiving sofosbuvir and daclatasvir ± RBV, and one patient treated with ledipasvir and sofosbuvir. Additionally, two patients (1.7%) treated with sofosbuvir and daclatasvir ± RBV developed a skin rash (Table 4).

**Table 4:** Adverse events of antiviral therapy in all patients.

	*n* (%)
Fatigue	5 (4.3)
Headache	4 (3.5)
Skin rash	2 (1.7)

*Note*: Data are presented as numbers (percentages).

## 4 DISCUSSION

Although the use of DAA is the current standard of care for patients with HCV infection in Saudi Arabia, data from the Eastern Province are limited. In this study, we attempted to fill this lack of knowledge and demonstrated that the DAAs are effective and safe. Overall, treatment with DAAs resulted in high rates of SVR12 among patients with HCV infection, regardless of the genotype, with minimal adverse events. The current study population had several unique characteristics, such as diverse HCV genotypes and different regimens of DAAs.

Our study's most significant finding is the high cure rate (98.3%), which, compared to other studies, demonstrates that variation in SVR rates can be attributed to several related factors. Our SVR12 rate was slightly higher than that recently reported by Hashim et al.,^14^ in their analysis of DAAs prescribed in private‐sector hospitals (97%). On the other hand, in patients with GT‐4, Babatin et al.^6^ reported an SVR rate of 100%, which is higher than the rate in our cohort, 50/52 (96.2%). This difference, although not significant, may be explained by the study's sample size, patient population, and treatment regimen. Furthermore, variations in patients' characteristics, viral genotypes, and treatment protocols may contribute to differences in SVR rates reported by others.

The association between hepatitis C infection and comorbid conditions such as NAFLD and dyslipidemia is significant, as demonstrated by the high prevalence of NAFLD (53%) and dyslipidemia (14.5%) in our patient population, and these conditions play a crucial role in the progression of liver fibrosis and impact the course and treatment of HCV infection. These findings are consistent with a previous study showing that 47.5% of patients with hepatitis C had fatty liver and that some patients had clinically significant fibrosis despite having normal liver enzyme levels.^15^ Both host and viral factors influence the development of hepatic steatosis.^16^ HCV may promote the accumulation of fat in the liver by interfering with the metabolism of lipids and glucose in liver cells, leading to increased insulin resistance and oxidative stress. In patients with HCV genotypes 3a and 1b, overexpression of core‐encoding sequences causes significant hepatic accumulations of triglycerides.^17^ The presence of NAFLD and dyslipidemia in patients with HCV infection may also impact the course and treatment of the infection and the overall risk of liver‐related complications. These findings suggest that patients with HCV infection and NAFLD require regular assessment for steatosis and fibrosis and long‐term follow‐up after achieving SVR, as the presence of steatosis plays a role in the progression of liver fibrosis.

The treatment of patients with HCV infection using DAAs has become less challenging, given the high rate of SVR in most genotypes, except GT‐3. Previous studies consistently showed effective DAA treatment across genotypes. For instance, a review reported 97%–100% SVR rates for treatment‐naïve GT‐1, 70%–100% for treatment‐experienced GT‐1, and 78%–97% for GT‐2.^12^ However, GT‐3 is challenging due to its association with severe liver disease, steatosis, and hepatocellular carcinoma development.^18^ Nevertheless, a recent meta‐analysis of noncirrhotic adults with genotype 3 infection treated for 8 weeks with glecaprevir/pibrentasvir showed an SVR12 rate of 99.2%, making GT‐3 no longer challenging.^19^ Notably, all our GT‐3 patients achieved SVR12 (100%); however, our study had only four GT‐3 patients, raising concerns about representativeness.

We included patients with different HCV genotypes than previous studies, which may have contributed to the lower prevalence of GT‐4 observed. In this study, 44.4% of patients with HCV were infected with GT‐4, while the rates of GT‐4 differ from other studies in Saudi Arabia. For example, a study of 1013 patients in Riyadh found that GT‐4 accounted for 60.0% of HCV cases,^20^ while in the Western Province of Saudi Arabia, 69.2% of HCV infections were due to GT‐4.^21^ A meta‐analysis of HCV genotypes in 18 studies from Saudi Arabia also found that 52.6% of HCV infections were due to GT‐4.^22^ Together, these studies confirmed the predominance and regional variability of HCV GT‐4 (60%–80%) in Saudi Arabia, but little data is available on the HCV‐4 subtype.^23^, ^24^ Furthermore, pangenotypic DAAs have some drawbacks, such as higher costs and the potential for the development of drug‐resistant strains of HCV. Therefore, further studies on resistance‐associated variants (RAVs) are needed.

The main limitations of the present study are the use of hospital‐based patients and selection bias. Moreover, the retrospective study design and data collected from a single center limit the generalizability of our findings. Because the study was conducted in a tertiary center, patients with mild diseases may have been excluded, and our study may be subject to reporting bias concerning treatment side effects. However, the key strengths of the study are the use of real‐life data, the variety of DAAs used, and the fact that the collected variables were extracted systematically under a predefined treatment protocol to minimize extraction errors and missing values. Furthermore, despite our retrospective study, our findings align with other studies on similar cohorts.

## 5 CONCLUSIONS

This study shows that DAAs were highly effective in treating HCV infection with few side effects. The effectiveness and safety of generic DAAs, HCV RAVs, and further studies on patients who fail treatment are necessary to identify predictors of antiviral therapy failure.

## AUTHOR CONTRIBUTIONS

**Mona H. Ismail**: Conceptualization; data curation; investigation; methodology; visualization; writing—original draft; writing—review and editing.

## ETHICS STATEMENT

King Fahad Hospital of the University is the hospital of Imam Abdulrahman Bin Faisal University. Thus, the Ethics Committee at Imam Abdulrahman bin Faisal University (IRB‐2019‐01‐373) issued this study's ethical approval, and the study was conducted according to the Declaration of Helsinki. Informed consent is waived due to the retrospective study design.

## TRANSPARENCY STATEMENT

The lead author Mona H. Ismail affirms that this manuscript is an honest, accurate, and transparent account of the study being reported; that no important aspects of the study have been omitted; and that any discrepancies from the study as planned (and, if relevant, registered) have been explained.

## Data Availability

Data are available upon request from the author.
